# Archeal tRNA meets biotechnology: From vaccines to genetic code expansion

**DOI:** 10.1016/j.jbc.2022.102755

**Published:** 2022-11-29

**Authors:** Lluís Ribas de Pouplana

**Affiliations:** 1 Institute for Research in Biomedicine, The Barcelona Institute of Science and Technology, Barcelona, Catalonia, Spain; 2 Catalan Institution for Research and Advanced Studies, Barcelona, Catalonia, Spain

**Keywords:** Biotechnology, Archaea, Genetic Code expansion, pyrrolysine, tRNA, pyrrolysine-tRNA synthetase, Pyl, pyrrolysine, PylRS, pyrrolysine-tRNA synthetase

## Abstract

Engineering new protein functionalities through the addition of noncoded amino acids is a major biotechnological endeavor that needs to overcome the natural firewalls that prevent misincorporation during protein synthesis. This field is in constant evolution driven by the discovery or design of new tools, many of which are based on archeal biology. In a recent article published in *JBC*, one such tool is characterized and its evolution studied, revealing unexpected details regarding the emergence of the universal genetic code machinery.

## Commentary

When Woese and Fox discovered the Archeal domain in 1977, their findings revolutionized microbiology (1). Archaea represented a third domain of living organisms, joining the ranks of bacteria and eukaryotes. Since its discovery, the archeal domain has grown steadily to include three different kingdoms and around 20 phyla in a very complex phylogenetic scenario, making single species determination challenging. These challenges extend to the analysis of the origin of Archaea in relation to bacteria and eukaryotes, as well as the nature of their last common ancestor. However, the obvious similarities between the fundamental genetic code machinery of archaea and eukaryotes are a strong argument for placing an archeal cell at the root of eukaryotic evolution.

The very same year that Archaea were discovered is considered by many to mark the birth of biotechnology, as this is the time when genetically engineered bacteria were first used to synthesize the recombinant protein therapeutic, somatostatin. With all probability, none of the fledging biotechnologists at the time would have been able to predict the impact that the discovery of Archaea would have on their field decades later.

Fast forward 42 years, to when the SARS-CoV-2 virus and the COVID-19 disease hit humanity. This led to one of the most impressive biotechnological efforts ever witnessed, the development and implementation of mRNA-based vaccines, at the heart of which was Archeal biology. More specifically, it is the modified nucleobase N1-methyl-pseudouridine, first discovered in archeal tRNAs that enables the mRNAs used in these vaccines to evade intrinsic immune responses and to be efficiently translated into viral antigens (2). In this, the two research avenues that started in 1977 (the discovery of Archaea and the advent of biotechnology) became inextricably linked.

Another surprising discovery brought about by the study of Archaea was that of genetically encoded pyrrolysine (Pyl) by the Krzycki laboratory in 2002 (3). The universal genetic code includes 20 amino acids, but many species expand that repertoire to 21 with selenocysteine or even 22 with Pyl. Incorporating Pyl as a coded amino acid for protein synthesis requires the development of a new tRNA (tRNA^Pyl^) and its cognate aminoacylating enzyme, pyrrolysine-tRNA synthetase (PylRS). However, this is not an easy task, as each new identity added to the genetic code has the potential to severely compromise its fidelity and efficiency through interferences with pre-existing components (4, 5).

Surprisingly, the PylRS•tRNA^Pyl^ pairs studied to date, when transplanted into other organisms, do not interfere with the fidelity of the host’s translation machinery. Consequently, researchers have exploited this property to expand the genetic code through the manipulation of PylRS (6). To achieve this goal, the substrate specificity of PylRS needed to be modified to recognize the amino acid of interest, and the anticodon of tRNA^Pyl^ changed to recognize a ‘free’ codon previously reassigned to the unnatural amino acid to be incorporated. The PylRS•tRNA^Pyl^ pair is nicely suited for this task because (a) the amino acid binding pocket of PylRS is large and can be adapted to different sidechains and (b) the anticodon sequence of tRNA^Pyl^ is not required for its aminoacylation by PylRS; thus, it can be mutated to recognize any codon of interest. Despite these advantages, achieving an efficient system for unnatural amino acid incorporation remains a daunting task.

As the interest in genetic code expansion grows, so does the search for new variants of the PylRS•tRNA^Pyl^ system. In 2014, a shorter form of the PylRS enzyme discovered in a group of methanogenic archaea (7) attracted attention because it did not interfere with the PylRS•tRNA^Pyl^ pairs in use at the time, thus permitting the incorporation of two different unnatural amino acids simultaneously (8, 9). In the article presented in this issue of *JBC* by the Tharp group, this variant of PylRS•tRNA^Pyl^ from *Ca. Methanomethylophilus alvus* (MaPylRS) is characterized in terms of its substrate recognition features and evolutionary history (10).

This report shows that, while MaPylRS is a relatively poor aminoacylating enzyme *in vitro*, its ability to translate engineered codons in *Escherichia coli* cells is at least 2-fold better than other PylRS variants. This interesting result illustrates the extent to which conditions within the cell may alter, or even reverse, the biochemical conclusions suggested by *in vitro* assays. In this particular case, the authors propose that the improved behavior of the enzyme *in vivo* may be due to its high solubility, and/or the fact that the cognate tRNA^Pyl^ for MaPylRS is better suited to function in the context of *E. coli* ribosomes.

The article also reveals interesting aspects of the PylRS•tRNA^Pyl^ evolution. A comprehensive homology search discovered that the number of species utilizing PylRS•tRNA^Pyl^ is much larger than previously assumed, extending beyond the methanogenic organisms that were initially considered the sole users of pyrrolysine. The authors also show that shorter forms of PylRS, such as MaPylRS, represent a derived form of the ancestral enzyme, which included additional domains that were lost secondarily. This conclusion is reinforced by the fact that most species encoding shortened forms of PylRS are endosymbiotic organisms undergoing processes of genome reduction. Possibly the most exciting conclusion reached by Guo *et al.* is that the emergence of PylRS is an ancient event that took place early in the evolution of Archaea or may even precede the appearance of LUCA. If this is indeed the case, then we may be forever left wondering why coded pyrrolysine was rejected by most species.

## Conflict of interest

The author declares that he has no conflicts of interest with the contents of this article
